# Migration of a Ureteral Double‐J Catheter From the Inferior Vena Cava to the Right Atrium With Concomitant Iliac Vein Thrombosis: Successful Retrieval and Anticoagulation Therapy—A Case Report

**DOI:** 10.1002/ccr3.73542

**Published:** 2026-09-16

**Authors:** Mostafa Ahmadi, Vafa Baradaran Rahimi, Sedegh Nazif

**Affiliations:** ^1^ Department of Cardiovascular Diseases, Faculty of Medicine Mashhad University of Medical Sciences Mashhad Iran; ^2^ Patient Safety Research Center, Clinical Research Institute Mashhad University of Medical Sciences Mashhad Iran

**Keywords:** catheter migration, double‐J catheter, inferior vena cava, right atrium, snare retrieval, thrombosis

## Abstract

We report the case of a 67‐year‐old male who underwent ureteral catheter placement for a right ureteral stone. Two months later, the catheter had migrated into the inferior vena cava and subsequently into the right atrium. The catheter was successfully removed using a snare via the right femoral vein.

## Key Clinical Message

Vascular migration of ureteral catheters into the heart is an extremely rare and life‐threatening complication. Prompt diagnosis through imaging and a multidisciplinary approach is crucial for safe catheter retrieval. Management should include both catheter retrieval and appropriate anticoagulation therapy.

## 1 Introduction

Ureteral catheters are widely used to relieve urinary obstruction. While common complications include infection, encrustation, and minor migration, migration into the venous system, particularly into the inferior vena cava and right atrium, is extremely rare and can lead to serious outcomes such as thrombosis, embolism, or cardiac injury [1, 2, 3]. Prompt diagnosis and effective management are therefore essential to prevent potentially life‐threatening complications. This case illustrates a successful interventional approach to retrieve a catheter that had migrated from the inferior vena cava to the right atrium [4, 5, 6]. It highlights the importance of managing the associated thrombus.

## 2 Case History/Examination

A 67‐year‐old male without any significant past medical history presented with right flank pain. Ultrasound showed a right ureteral stone, and he subsequently underwent ureteroscopy, and a double‐J catheter was inserted without immediate complications.

Two months later, the patient returned for catheter removal. Initially, the patient was evaluated by the urology team, and one attempt at conventional urological retrieval was performed; however, the catheter could not be successfully retrieved. The patient was subsequently referred to our center for further management.

## 3 Investigations and Treatment

**FIGURE 1:**
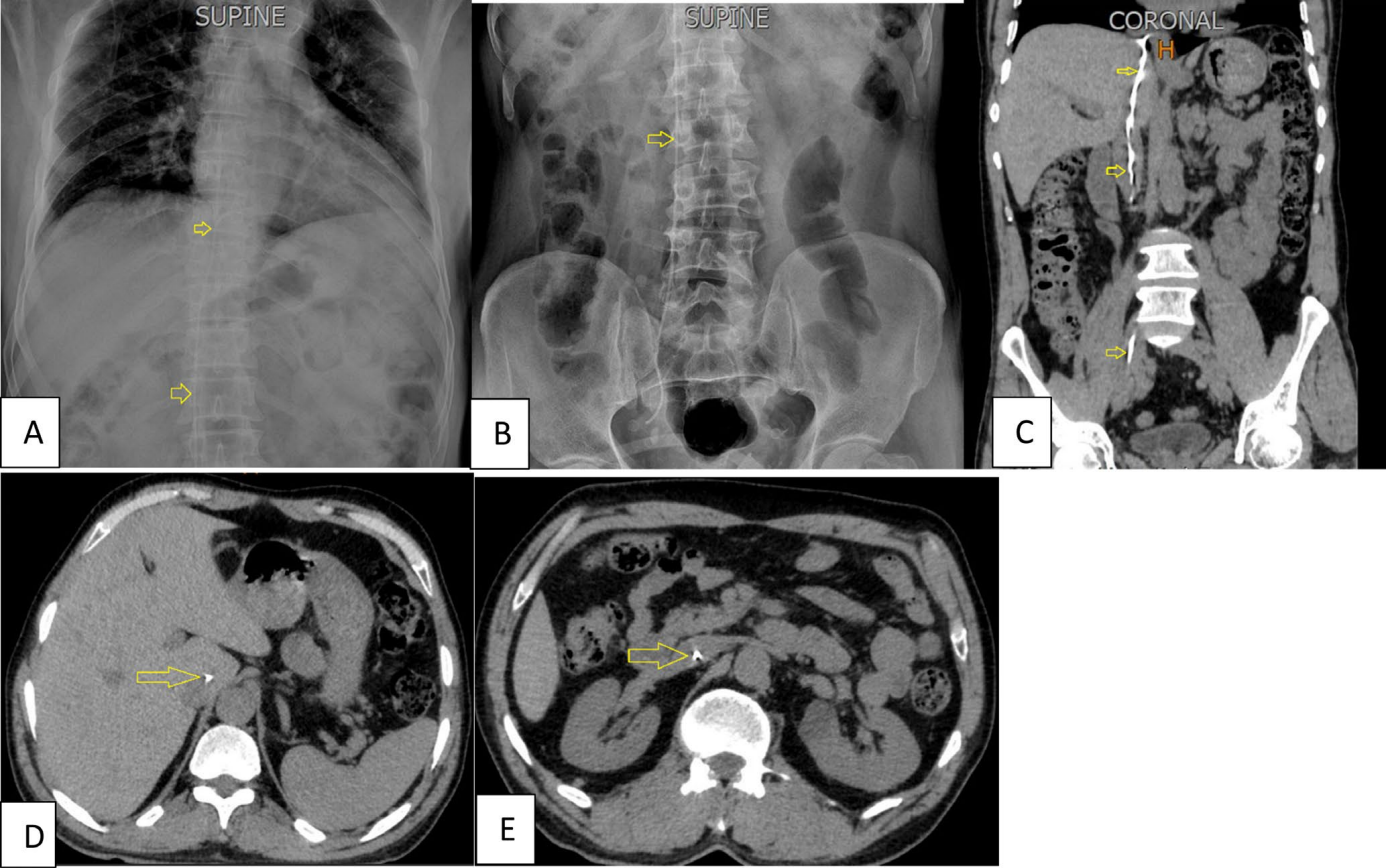
(A, B) The double‐J catheter has migrated into the IVC, as demonstrated on radiographs. The catheter tip is visualized in the right atrium. (C–E) The double‐J catheter was shown in coronal and axial views of a CT scan.

Imaging studies, including X‐ray and CT scans, revealed the catheter had migrated into the inferior vena cava (IVC) and subsequently into the right atrium (Figure 1A–E). In Figure 1A,C, the catheter tip is visualized in the right atrium.

**FIGURE 2:**
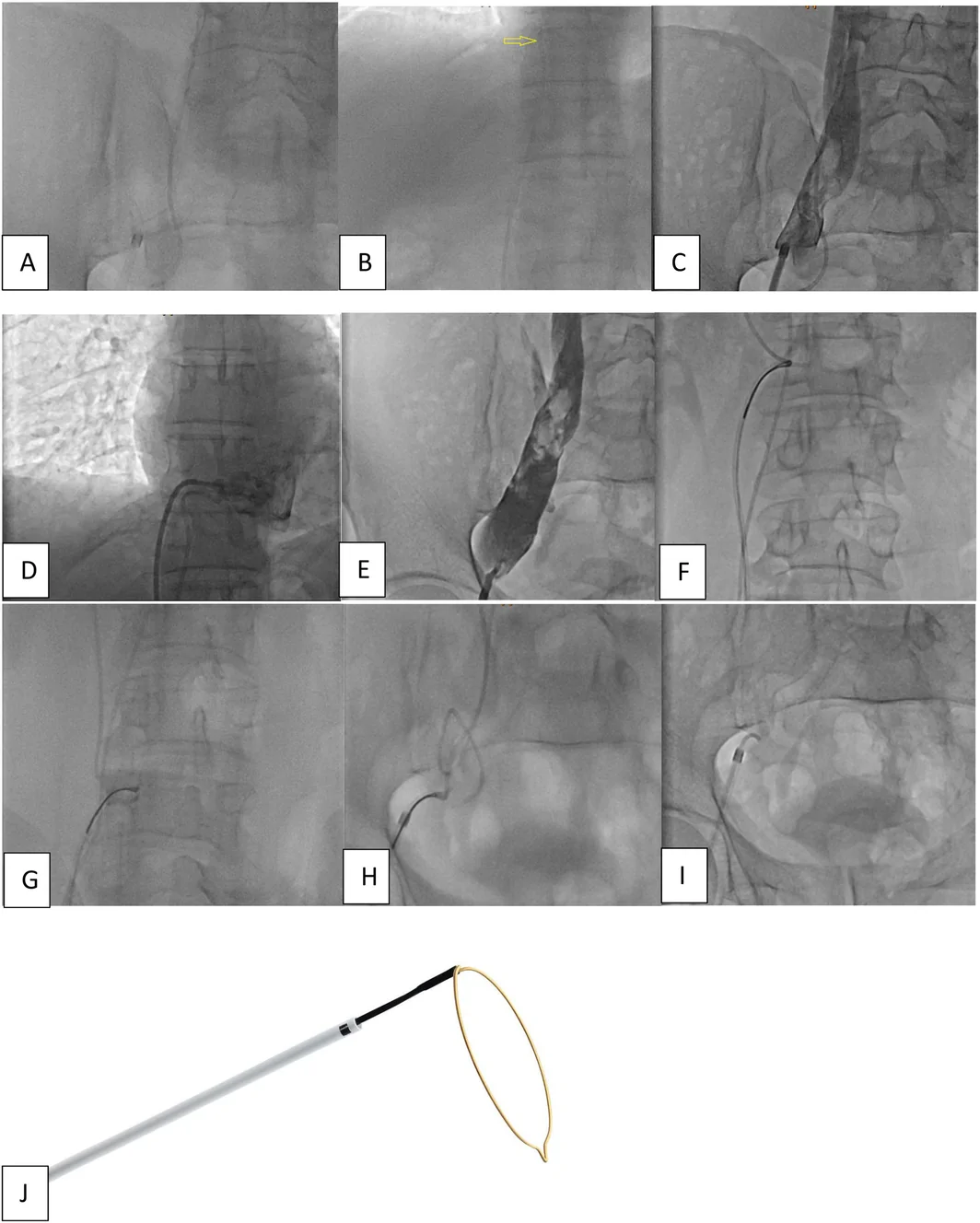
Sequential images demonstrating the steps of angiography. (A, B) A double‐J catheter is seen in the IVC, and the tip of the catheter in the RA (yellow arrow). (C) Injection in the common iliac artery and presence of the double‐J catheter in the IVC. (D) Injection in the RA cavity and presence of the tip of the catheter in the RA. (E) A large thrombus is seen in the common iliac vein. (F–I) Images of snaring and removal of the double‐J catheter via the right femoral vein. (J) ONE Snare Endovascular System (Merit).

Venography was performed. It was demonstrated that the catheter had completely migrated out of the urinary tract and was located entirely within the venous system, with no residual portion remaining in the ureter or urinary bladder (Figure 2A–D). A large thrombus was also noted to have formed in the common iliac vein, surrounding the catheter (Figure 2E). Based on these findings, endovascular retrieval was planned.

Under fluoroscopic guidance, the migrated double‐J catheter was successfully retrieved from the right atrial cavity (Figure 2F–I). A 6‐Fr sheath was inserted via the right common femoral vein. A 10‐mm ONE Snare Endovascular System (Merit Medical, South Jordan, UT, USA) was advanced to capture the migrated catheter (Figure 2J). After vascular access, intravenous unfractionated heparin (100 U/kg) was administered. Activated clotting time was not monitored because of the short procedural duration. The catheter and attached organized thrombus were retrieved en bloc without procedural complications.

Following snare deployment, confirmatory fluoroscopy was performed, demonstrating complete catheter extraction with no residual intravascular fragments. The retrieved double‐J catheter was also carefully inspected ex vivo, confirming its structural integrity and the absence of any missing pieces. The patient subsequently received a heparin infusion to manage the associated thrombosis.

## 4 Conclusion and Results

After removal of the catheter from the IVC, a new double‐J stent was reinserted in the correct anatomical position. The patient remained clinically stable throughout the postoperative course and was discharged without further complications.

## 5 Discussion

Migration of ureteral double‐J catheters into the IVC is an extremely rare complication. The presence of a foreign body, such as a catheter, within the venous system can cause endothelial injury and stasis, predisposing to thrombus formation. This complication significantly increases the risk of pulmonary embolism and other vascular events.

Our case is consistent with these reports in terms of the rarity of IVC migration and the need for specialized retrieval (Table 1). A 33‐year‐old pregnant woman experienced migration of a double‐J stent, which had traveled from the right iliac vein into the inferior vena cava and eventually reached the right atrium. She was transferred to the vascular surgery unit, where the device was removed using an endovascular approach via femoral vein puncture. Under fluoroscopic guidance, a curved guidewire was advanced through the vena cava to the right atrium, allowing successful retrieval of the endoprosthesis [4]. Another case involved a 53‐year‐old male who experienced migration of a double‐J catheter into the IVC after left pyelolithotomy. Endoscopic retrieval under C‐arm guidance was performed successfully [5].

**TABLE 1:** Three cases with migration of the ureteral catheter.

Patient age/sex	Reason for catheterization	Migration location	Retrieval method	Outcome	Refs
33 years old/female	Pregnancy‐related ureteral obstruction	IVC → Right atrium	Snare via the femoral vein	Successful	[4]
53 years old/male	Post‐pyelolithotomy	IVC	Endoscopic with C‐arm	Successful	[5]
67 years old/male	Right ureteral stone	IVC → Right atrium	Snare via the femoral vein	Successful	This study

What makes our case particularly noteworthy is the concomitant iliac vein thrombosis, which highlights an additional layer of complexity and risk. Unlike some prior cases, conventional urological methods failed in our patient, and the catheter was successfully removed using interventional cardiology techniques via femoral vein snaring. This highlights the critical importance of a multidisciplinary approach when managing this rare but potentially life‐threatening complication, especially when the catheter has reached a cardiac chamber and has caused thrombosis. The successful retrieval of the catheter followed by prompt anticoagulation therapy underscores a crucial aspect of managing such cases.

It should be noted that pulmonary embolism is a potential complication during retrieval of catheter‐associated thrombi, particularly when fresh or mobile thrombus is present. In our case, preprocedural CT and fluoroscopic findings suggested that the thrombus was chronic and organized rather than fresh or friable. The thrombus appeared firmly attached to the catheter, and there were no features suggesting a large mobile thrombus with a high risk of embolization. Therefore, prophylactic placement of an IVC filter was not considered necessary. No clinical or imaging evidence of pulmonary embolism was observed during or after the procedure.

This case highlights the importance of early cross‐sectional imaging in patients with suspected catheter migration. Confirmation of complete intravascular migration can facilitate prompt endovascular management and may prevent unnecessary repeated urological retrieval attempts.

## 6 Conclusion

Vascular migration of ureteral catheters, while rare, can lead to serious complications. Early recognition, careful imaging, and a multidisciplinary approach are critical. Interventional techniques, including femoral vein snaring, can achieve safe retrieval and favorable patient outcomes.

## Author Contributions

**Mostafa Ahmadi:** conceptualization, supervision, investigation, project administration, resources, writing – review and editing, validation. **Vafa Baradaran Rahimi:** writing – review and editing, visualization, supervision, validation. **Sedegh Nazif:** investigation, writing – original draft, writing – review and editing, project administration.

## Funding

The authors have nothing to report.

## Ethics Statement

The authors have nothing to report.

## Consent

Written informed consent of the patient was obtained for publication.

## Conflicts of Interest

The authors declare no conflicts of interest.

## Data Availability

The data that support the findings of this study are available from the corresponding author upon reasonable request.
